# Supplementary material for: Synthesis of Cellulose–Poly(Acrylic Acid) Using Sugarcane Bagasse Extracted Cellulose Fibres for the Removal of Heavy Metal Ions
Source: Int J Mol Sci. 2023 May 18;24(10):8922. doi: 10.3390/ijms24108922 (PMC10219175; doi:10.3390/ijms24108922)
Supplement: Supplementary file 1 [file ijms-24-08922-s001.zip › ijms-2335379-supplementary.pdf]

Supplementary information

Synthesis of cellulose–poly(acrylic acid) using sugarcane bagasse extracted cellulose fibres for the removal of heavy metal ions

Fuchao Li, Zhemin Xie, Jianfeng Wen, Tao Tang, Li Jiang, Guanghui Hu* and Ming Li*

College of Science & Key Laboratory of Low-dimensional Structural Physics and Application, Education Department of Guangxi Zhuang Autonomous Region, Guilin University of Technology, Guilin 541004, China

*Correspondence: guanghui@glut.edu.cn (G. Hu), 2006017@glut.edu.cn (M. Li).

Swelling Capacity

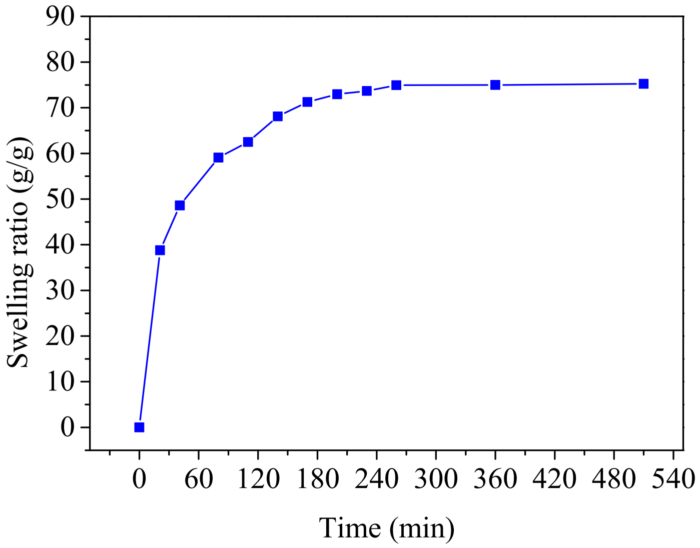

Figure S1. Swelling ratios of CE-PAANa

The swelling ratio of the sample was measured by immersing 0.2 g of hydrogel into 200 mL of water to reach equilibrium in a beaker (20 °C), and the weight of the

swollen hydrogel was measured after removing excess water which had not penetrated into the hydrogel by a mesh sieve at different contact time until equilibrium. The swelling ratio of hydrogels can be calculated as SR = $(W_s - W_d)/W_d$, where W_s and W_d represent the weights of swollen hydrogels and dry hydrogels, respectively.

The CE-PAANa had swelling capacity of 75 g/g in distilled water, as shown in Fig. S2. As shown in Table S1, the low swelling ratio of CE-PAANa is due to the access to a large amount of cellulose. This is because the addition of CE leads to a shrinkage of the hydrogel pores, which slows the entry of water[1, 2].

Table S1. The swelling capacity in distilled water reported for some grafted PAA

**Materials**	**Equilibrium swelling ratio (g·g−1)**	**Ref.**
Pb (II) P(AANa-co-AM)/GO	282.2	[3]
HEC-g-P（AA-co-AM）	240	[4]
WS-g-P（AA-co-AM）	200	[5]
CE-PAANa	75	This study

Table S2. Gel Permeation Chromatography (GPC) test results for cellulose.

Sample	Method	Retention time (min)	GPC M_n (g/mol)	GPC M_w (g/mol)	GPC M_z (g/mol)	GPC M_{z+1} (g/mol)	GPC M_η (g/mol)	Dispersity M_w/M_n
CE	DMSO-PMMA	8.6471	6678	9599	12973	16415	9134	1.4374

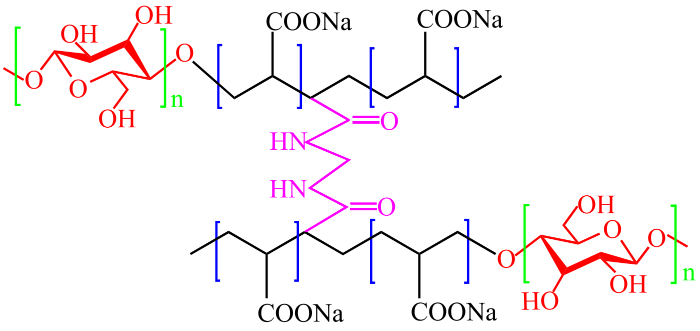

Figure S2. Schematic diagram of the structure of CE-PAANa

References

1. Chen, Y.; Li, Q.; Li, Y.; Zhang, Q.; Huang, J.; Wu, Q.; Wang, S., Fabrication of Cellulose Nanocrystal-g-Poly(Acrylic Acid-Co-Acrylamide) Aerogels for Efficient Pb(II) Removal. In *Polymers*, 2020; Vol. 12.
2. Yu, F.; Yang, P.; Yang, Z.; Zhang, X.; Ma, J., Double-network hydrogel adsorbents for environmental applications. *Chemical Engineering Journal* **2021,** 426, 131900.
3. He, S.; Zhang, F.; Cheng, S.; Wang, W., Synthesis of Sodium Acrylate and Acrylamide Copolymer/GO Hydrogels and Their Effective Adsorption for Pb2+ and Cd2+. *ACS Sustainable Chemistry & Engineering* **2016,** 4, (7), 3948-3959.
4. Sultan, M.; Nagieb, Z. A.; El-Masry, H. M.; Taha, G. M., Physically-crosslinked hydroxyethyl cellulose-g-poly (acrylic acid-co-acrylamide)-Fe3+/silver nanoparticles for water disinfection and enhanced adsorption of basic methylene blue dye. *International Journal of Biological Macromolecules* **2022,** 196, 180-193.
5. Song, J.; Zhao, H.; Zhao, G.; Xiang, Y.; Liu, Y., Novel Semi-IPN Nanocomposites with Functions of both Nutrient Slow-Release and Water Retention. 1. Microscopic Structure, Water Absorbency, and Degradation Performance. *J. Agric. Food. Chem.* **2019,** 67, (27), 7587-7597.
